# The missclassification of obesity affects the course of migraine

**DOI:** 10.1186/s10194-018-0895-6

**Published:** 2018-08-03

**Authors:** Laura Di Renzo, Andrea Cammarano, Antonino De Lorenzo

**Affiliations:** 1 Section of Clinical Nutrition and Nutrigenomic, Department of Biomedicine and Prevention, University of Rome Tor Vergata, Via Montpellier 1, 00133 Rome, Italy; 2 Department of Experimental Medicine and Surgery, University of Rome Tor Vergata, Via Montpellier 1, 00133 Rome, Italy

Migraine, a primary headache disorder, with unilateral and recurrent attacks of moderate to severe pain intensity, is the main cause of disability in life. Migraine is manifested with concomitant photophobia, phonophobia and nausea and is associated with an increased risk of chronic degenerative diseases, cardiopathies, psychiatric diseases, neurological disorders, respiratory disorders, hypertension, diabetes, and obesity. The prevalence of migraine, either episodic or chronic, higher in obese individuals may depend on a common etiological factor. Moreover, episodic migraine in obese subjects are influenced by age, race, and sex [1]. Both conditions are linked to the inflammatory processes, through the release of proinflammatory cytokines and neuropeptides. Indeed, a relationship between inflammation and predisposition to migraine has been hypothesized.

An high inflammatory response promotes the sensitization of central neurons to harmful and non-harmful stimuli, with an increased risk of progression of migraine, and the increase in body fat mass of the obesity state can intensify the neurovascular inflammatory response in migraine, increasing headache and its frequency [2, 3].

Although the headache is stronger in obese or overweight subjects, the number of epidemiological studies on obesity and headaches does not currently allow to determine the causal link in this relationship. The paradox seems to exist for those subjects with a body mass index (BMI) in the normal range, but with recurrent headaches with increasing intensity and frequency. Moreover, literature data shows that the risk of migraine is increased in obese and underweight individuals. In order to explain the paradox it is important to understand which tools and parameters are used to diagnose obesity [4].

The parameters currently adopted by the Ministries of Health to diagnose obesity are old, limited and often unfit to discover hidden fat. Just think that to date, the most used parameter to define and classify overweight and obesity in adults is the Body Mass Index (BMI), the ratio between weight in kilograms and height in meters squared (kg/m^2^), that does not distinguish between total body fat (TBFat) and total body lean (TBLean), or bone mass. Moreover, the cut-offs of BMI have been chosen arbitrary, it does not consider age, gender and ethnicity.

Therefore, the use of BMI as an index of PBF and diagnosis of obesity may be inaccurate [4].

A lot of data support the idea that to overcome misclassifications of obese status, direct measurements of PBF, using dual energy x-ray absorptiometry (DXA), appears to be a better tool, respect all the anthropometric evaluations. In fact, in people with normal BMI we demonstrated a wide range of percentage of body fat (PBF), ranging from 5.6 to 31.2% in men and from 4.6 to 51.1% among women. Current research suggests that obesity cut-off points of PBF are in the 23%–25% range in men and 30%–33%–35% range in women.

Anyway, to avoid erroneous classifications, the diagnosis and treatment of obesity can not be separated from a careful general and nutritional history, from the objective examination, from the measurement of biochemical and hormonal parameters, from the measurement of energy expenditure at rest and, above all, from the evaluation of the body composition.

If we analyze the definition of obesity of the World Health Organization (WHO), which identifies obesity as “a pathological condition in which body fat is increased to an extent in which health and well-being are impaired”, we can affirm that it is defined precisely by the expansion of adipose tissue, rather than defining it solely on the basis of the increase in body weight [5]. It is in fact in this direction that the research has been oriented in the last decade, focusing precisely on the “adipose tissue”. Furthermore, obesity is characterized by a state of chronic low-grade inflammation of the adipose tissue, which acts as a neuroendocrine organ, producing multiple molecules involved in energy homeostasis and inflammation, via the secretion of inflammatory cytokines. The adipocytes produce and secrete a large number of molecules of a protein nature, collectively called “adipokine”. More than 50 are currently identified, but the list is growing day by day, including: cytokines, such as tumor necrosis factor TNF-α, interleukins (IL-1, IL-6, IL-10), Transforming Growth Factor-β (TGF-β), leptin, resistin, adiponectin, visfatin, chemokines, such as IL-8, Monocyte Chemoattractive Protein-1 (MCP-1), alternative complement system proteins, such as adipsin, hemostatic proteins (Plasminogen Activator Inhibitor-1 (PAI-1), proteins involved in the regulation of arterial pressure (angiotensinogen), angiogenic proteins (Vascular Endothelial Growth Factor (VEGF), neurotrophins (Nerve Growth Factor (NGF), acute phase proteins (haptoglobin, Serum Amyloid-A (SAA) metallothionin.

These adipocytokines may be involved in the correlation between obesity and migraine [6].

To this list can be added the calcitonin gene- related peptide (CGRP) that could represent another link between obesity and migraine. CGRP is a neurotransmitter produced in peripheral sensory neurons and numerous sites of the central nervous system, able to promote immune and inflammatory responses, secondary to vasodilatation, that is increased during acute migraine attacks [7]. Moreover, CGRP possesses diverse biological effects, including the modulation of sensations, learning and memory, food intake, autonomic functions, skeletal muscle motility, and gastrointestinal tract functions.

Furthermore, it has been shown an increase in CGRP levels in obese subjects, recombined with a CGRP/sensory nerve activity at an early stage in the metabolic syndrome [8].

Therefore, the possibility of evaluating body composition, fat composition and distribution, lipid and glucose metabolism, low-grade inflammation, and genetic makeup, allowed to identify the real phenotype of obesity, with the possibility of personalized treatment [4].

A key in the study of obesity and related diseases has been offered by our recognition of the Normal Weight Obese Syndrome (NWO) syndrome, which is a typical syndrome with a chronic low-grade inflammation. NWO was defined as subjects with a normal BMI (18.5–24.9 kg/m2) and an excess in PBF, defined by the highest sex specific tertiles of PBF (23.1% in men and 30–33.3% in women), with a reduced muscle mass and an increase in fat mass, predisposing to the risk of chronic degenerative diseases. The cardiovascular disease risk indexes are significantly elevated, and some inflammatory parameters were altered. The increase of TNF-α, IL-1α, IL -1β, IL -6, IL -8 supports the hypothesis that the syndrome is characterized by an early inflammatory state. Moreover, single nucleotide polymorphisms in some of the genes for the mediators of inflammation discriminate the NWO subjects from healthy people [4, 9].

Since it has been demonstrated an association between migraine and proteins mainly secreted by adipose tissue capable of contributing to energy homeostasis and neuroinflammatory processes, it is important to find a further factor that may explain the exacerbation of headaches in non predictable situation [10].

The microbiota could be the connection between inflammation, obesity and headache.

Gut microbiota plays an important role in host physiology. Indirect effects of the gut microbiota on the innate immune system can result in alterations in the circulating levels of pro- and anti-inflammatory cytokines that directly affect brain function, especially areas such as the hypothalamus, where IL-1 and IL-6 provide a potent release of corticotrophin releasing hormone (CRH), a regulator of the hypothalamic-pituitary-adrenal axis (HPA), which itself provides another bidirectional route of communication.

Dysbiosis and dysregulation of the microbiota-gut brain axis have been implicated in gastrointestinal disorders, inflammatory diseases, metabolic disorders and neurological disorders, such as general anxiety disorder, depression and autism. Moreover, a higher prevalence of headache was found in individuals with gastrointestinal (GI) disorders, as reflux, diarrhoea, constipation and nausea [11].

The vagus nerve has both efferent and afferent divisions, and plays a fundamental role in enabling signals from brain to gut and vice versa. Exposure to stress, defined as “a state of threatened homeostasis”, can impair a number of functions and induce GI and mental disorders, such as anxiety.

A randomized sham-controlled trial with noninvasive vagus nerve stimulation (nVNS) demonstrated effective benefits as pain relief in episodic migrainers, reversing the elevation in extracellular glutamate, and facilitating central inhibition in the trigeminovascular system [12].

As anxiety disorders are strongly associated with migraine, obese subjects are prone to anxiety and chronic, disabling migraine, the inflammatory status affected the equilibrium of the psycobioma, this full circle coul be significant for the potential implications regarding the pathogenetic link of these conditions.

It has been highlighted that probiotics produce a variety of neurochemicals, analogues of hormones involved in mood and behaviour, and play a therapeutic potential deriving from modulation of gut composition in such conditions.

Therfore we investigated the role of psychobiotics in NWO subjects. In our previous study, NWO subjects highlighted body dissatisfaction and drive for thinness [13]. Moreover, they are prone to anxiety. In these conditions, we observed a significant improvement of the orocaecal transit time and gastrointestinal symptoms after three-week intake of selected psychobiotics, that are able in the meantime to modulate body composition, and psychopathological scores of NWO subjects [14, 15].

In obese subjects, significant improvements in migraine frequency were demonstrated after weight loss [3] than probiotics treatment [16].

Restoring the integrity of the intestinal barrier with probiotics such disorders of the brain, involving depression, anxiety, frequency and intensity of migraine attack, have been reduced, with a bidirectional modulation of gut microbiota and brain function, through the decrease of inflammatory reactions.

In conclusion, emerging research data suggest the use of probiotics could be a suitable therapeutic intervention for depression and related disorders, involved in the regulation of neuroinflammation, neuroendocrine stress response. Further large-scale randomized placebo-controlled trials to confirm efficacy and safety of probiotics in patients with migraine headache before arriving at definitive conclusions [17].

## References

[1] Peterlin BL, Rosso AL, Williams MA, Rosenberg JR, Haythornthwaite JA, Merikangas KR, Gottesman RF, Bond DS, He JP (2013). Zonderman AB episodic migraine and obesity and the influence of age, race, and sex. Neurology.

[2] Andreeva VA, Galan P, Julia C, Fezeu L, Hercberg S, Kesse-Guyot E. A systematic literature review of observational studies of the bidirectional association between metabolic syndrome and migraine. Diabetes Metab. 2017. 10.1016/j.diabet.2017.12.004 10.1016/j.diabet.2017.12.004 29336986

[3] Bond DS, Roth J, Nash JM, Wing RR (2011). Migraine and obesity: epidemiology, possible mechanisms and the potential role of weight loss treatment. Obes Rev.

[4] De Lorenzo A, Soldati L, Sarlo F, Calvani M, Di Lorenzo N, Di Renzo L (2016). New obesity classification criteria as a tool for bariatric surgery indication. World J Gastroenterol.

[5] World Health Organization (2000). Obesity: Preventing and Managing the Global Epidemic. Report Series 894 on a WHO Consultation on Obesity.

[6] Dominguez C, Vieites-Prado A, Perez-Mato M, Sobrino T, Rodriguez-Osorio X, Lopez A, Campos F, Martinez F, Castillo J, Leira R (2018). Role of adipocytokines in thempathophysiology of migraine: a cross-sectional study. Cephalalgia.

[7] Russell FA, King R, Smillie SJ, Kodji X, Brain SD (2014). Calcitonin gene-related peptide: physiology and pathophysiology. Physiol Rev.

[8] Zelissen PM, Koppeschaar HP, Lips CJ, Hackeng WH (1991). Calcitonin gene-related peptide in human obesity. Peptides.

[9] Di Renzo L, Bianchi A, Saraceno R, Calabrese V, Cornelius C, Iacopino L, Chimenti S, De Lorenzo A (2012). 174G/C IL-6 gene promoter polymorphism predicts therapeutic response to TNF-α blockers. Pharmacogenet Genomics.

[10] Lénárt N, Brough D, Dénes Á (2016). Inflammasomes link vascular disease with neuroinflammation and brain disorders. J Cereb Blood Flow Metab.

[11] Soldati L, Di Renzo L, Jirillo E, Ascierto PA, Marincola FM, De Lorenzo A (2018). The influence of diet on anti-cancer immune responsiveness. J Transl Med.

[12] Tassorelli C, Grazzi L, de Tommaso M, Pierangeli G, Martelletti P, Rainero I, Dorlas S, Geppetti P, Ambrosini A, Sarchielli P, Liebler E, Barbanti P; PRESTO Study Group. Noninvasive vagus nerve stimulation as acute therapy for migraine: The randomized PRESTO study. Neurology. 2018. 10.1212/WNL.0000000000005857 10.1212/WNL.0000000000005857 PMC6070381 29907608

[13] Di Renzo L, Tyndall E, Gualtieri P, Carboni C, Valente R, Ciani AS, Tonini MG, De Lorenzo A (2016). Association of body composition and eating behavior in the normal weight obese syndrome. Eat Weight Disord.

[14] De Lorenzo A, Costacurta M, Merra G, Gualtieri P, Cioccoloni G, Marchetti M, Varvaras D, Docimo R, Di Renzo L (2017). Can psychobiotics intake modulate psychological profile and body composition of women affected by normal weight obese syndrome and obesity? A double blind randomized clinical trial. J Transl Med.

[15] Colica C, Avolio E, Bollero P, Costa de Miranda R, Ferraro S, Sinibaldi Salimei P, De Lorenzo A, Di Renzo L (2017). Evidences of a New Psychobiotic Formulation on Body Composition and Anxiety. Mediators Inflamm.

[16] Dai YJ, Wang HY, Wang XJ, Kaye AD, Sun YH (2017). Potential beneficial effects of probiotics on human migraine headache: a literature review. Pain Physician.

[17] de Roos NM, van Hemert S, Rovers JMP, Smits MG, Witteman BJM (2017). The effects of a multispecies probiotic on migraine and markers of intestinal permeability-results of a randomized placebo-controlled study. Eur J Clin Nutr.

